# Follow-Up Assessment of Intracranial Aneurysms Treated with Endovascular Coiling: Comparison of Compressed Sensing and Parallel Imaging Time-of-Flight Magnetic Resonance Angiography

**DOI:** 10.3390/tomography8030133

**Published:** 2022-06-18

**Authors:** Gianfranco Vornetti, Fiorina Bartiromo, Francesco Toni, Massimo Dall’Olio, Mario Cirillo, Peter Speier, Ciro Princiotta, Michaela Schmidt, Caterina Tonon, Domenico Zacà, Raffaele Lodi, Luigi Cirillo

**Affiliations:** 1 IRCCS Istituto delle Scienze Neurologiche di Bologna, UOC di Neuroradiologia, 40139 Bologna, Italy; gianfranco.vornetti@gmail.com (G.V.);; m.dallolio@isnb.it (M.D.); c.princiotta@isnb.it (C.P.); 2 IRCCS Istituto delle Scienze Neurologiche di Bologna, Programma Neuroimmagini Funzionali e Molecolari, 40139 Bologna, Italy; fiorina.bartiromo2@unibo.it (F.B.); caterina.tonon@unibo.it (C.T.); raffaele.lodi@unibo.it (R.L.); 3 IRCCS Istituto delle Scienze Neurologiche di Bologna, Programma di Neuroradiologia con Tecniche ad Elevata Complessità, 40139 Bologna, Italy; francesco.toni@isnb.it; 4 Dipartimento di Scienze Mediche e Chirurgiche Avanzate, Università della Campania "Luigi Vanvitelli", 81100 Napoli, Italy; mario.cirillo@unicampania.it; 5 Siemens Healthineers, 91052 Erlangen, Germany; peter.speier@siemens-healthineers.com (P.S.); michaela.schmidt@siemens-healthineers.com (M.S.); domenico.zaca@siemens-healthineers.com (D.Z.); 6 Dipartimento di Scienze Biomediche e Neuromotorie, Università di Bologna, 40138 Bologna, Italy

**Keywords:** magnetic resonance angiography, compressed sensing, intracranial aneurysm, coil embolization

## Abstract

The aim of our study was to compare compressed sensing (CS) time-of-flight (TOF) magnetic resonance angiography (MRA) with parallel imaging (PI) TOF MRA in the evaluation of patients with intracranial aneurysms treated with coil embolization or stent-assisted coiling. We enrolled 22 patients who underwent follow-up imaging after intracranial aneurysm coil embolization. All patients underwent both PI TOF and CS TOF MRA during the same examination. Image evaluation aimed to compare the performance of CS to PI TOF MRA in determining the degree of aneurysm occlusion, as well as the depiction of parent vessel and vessels adjacent to the aneurysm dome. The reference standard for the evaluation of aneurysm occlusion was PI TOF MRA. The inter-modality agreement between CS and PI TOF MRA in the evaluation of aneurysm occlusion was almost perfect (κ  =  0.98, *p*  <  0.001) and the overall inter-rater agreement was substantial (κ  =  0.70, *p*  <  0.001). The visualization of aneurysm parent vessel in CS TOF images compared with PI TOF images was evaluated to be better in 11.4%, equal in 86.4%, and worse in 2.3%. CS TOF MRA, with almost 70% scan time reduction with respect to PI TOF MRA, yields comparable results for assessing the occlusion status of coiled intracranial aneurysms. Short scan times increase patient comfort, reduce the risk of motion artifacts, and increase patient throughput, with a resulting reduction in costs. CS TOF MRA may therefore be a potential replacement for PI TOF MRA as a first-line follow-up examination in patients with intracranial aneurysms treated with coil embolization.

## 1. Introduction

The global prevalence of intracranial aneurysms is approximately 2–3% [1,2], with aneurysm-related morbidity and mortality primarily arising from their rupture. Treatment strategies aim to exclude the aneurysm from the intracranial circulation to eliminate the risk of bleeding (or re-bleeding). Endovascular treatment of intracranial aneurysms is the current standard of care [3] and comprises coil occlusion, with or without stent assistance, as well as parent vessel reconstruction using flow-diverting stents. However, treated aneurysms must be followed over time to ensure durable occlusion since recurrences occur in approximately 20% of endovascularly treated patients, leading to a need for retreatment in approximately 9% of all cases [4]. While digital subtraction angiography (DSA) is the reference standard for evaluating aneurysm recurrence, this invasive technique exposes patients to risks such as ionizing radiation, contrast nephrotoxicity, and cerebral thromboembolism [5,6]. These risks accumulate because stability of aneurysm occlusion must be confirmed over time, requiring repeated follow-ups.

Magnetic resonance angiography (MRA) is a non-invasive technique that can be employed for the follow-up of endovascularly treated aneurysms, eliminating some of the risks associated with serial DSA. Several studies showed that MRA has high sensitivity and specificity for identifying recanalization of aneurysms treated with coil embolization, with or without stent assistance [7,8,9,10,11,12,13,14]. Traditionally, both contrast-enhanced (CE) MRA and time-of-flight (TOF) MRA have been used for aneurysm follow-up. TOF MRA has the additional advantage of not requiring intravenous contrast agent administration, while showing comparable diagnostic accuracy to CE MRA, and is therefore recommended as the routine follow-up method for the detection of residual flow in intracranial aneurysms treated with endovascular coil occlusion [7,15,16,17].

Nonetheless, TOF MRA is a time-consuming technique, which reduces patient throughput and makes it susceptible to motion artifacts, limiting its applicability to uncooperative patients. Parallel imaging (PI) is commonly used to reduce scan times of TOF MRA, but acceleration factors are usually not higher than 2 or 3 as further reduction in phase-encoding steps will rapidly increase noise and aliasing, resulting in typical scan times not shorter than 5–6 minutes [18]. Compressed sensing (CS) is a mathematical framework that reconstructs data from incoherent undersampled measurements. CS has been applied to MRI to achieve higher acceleration factors by k-space undersampling, through exploitation of the underlying sparsity in the appropriate transform domain [19,20]. TOF MRA is a good candidate for CS based acceleration, as angiographic images feature sparse vessels with high signal over a well-suppressed background [21].

Recent studies showed that CS TOF MRA has comparable sensitivity and specificity to PI TOF MRA in the detection of a variety of cerebrovascular pathologies, including aneurysms, stenosis and arteriovenous shunts, while allowing a 2- to 3-fold reduction in scan time [21,22,23,24,25]. However, no data are available on the diagnostic accuracy of CS TOF MRA in the follow-up of endovascularly treated aneurysms. Here, we show a comparison of CS and PI TOF MRA in the evaluation of patients with intracranial aneurysms treated with coil embolization or stent-assisted coiling.

## 2. Materials and Methods

Between May and September 2020, we evaluated all patients who underwent follow-up imaging at our institution for a treated intracranial aneurysm (*n* = 32). Our follow-up protocol for patients with endovascularly treated intracranial aneurysm consists of an MRA before hospital discharge and then at 3-month intervals for the first year. After 12 months, if no recanalization is observed, patents undergo yearly follow-up examinations. We enrolled in this study all patients (*n* = 22) who underwent endovascular treatment of the aneurysm with either coil embolization or stent-assisted coiling. Patients treated with flow-diverting stents (*n* = 10) were excluded from the study.

The reference standard for the evaluation of aneurysm occlusion was PI TOF MRA.

This study was carried out in accordance with ethical standards as set out in the Declaration of Helsinki and Directive 2005/28/EC and approved by the institutional review board (IRCCS Istituto delle Scienze Neurologiche di Bologna). The use of CS TOF MRA was approved by the Local Ethics Committee, with the Prot. N. 82/2019/Sper/AOUbo.

### 2.1. Image Acquisition

All patients underwent both a standard PI and a prototype CS TOF MRA sequence during the same examination. Imaging was performed with a 3T MR scanner (MAGNETOM Skyra, Siemens Healthcare, Erlangen, Germany) with a 64-channel head/neck coil. The acceleration factor for the prototype CS TOF MRA was 10, with incoherent sampling using variable-density Poisson sampling, as described in a previous study [21]. Parameters for the CS TOF MRA sequence were TR 21 ms, TE 3.49 ms and flip angle 18°. In total, 4 slabs were acquired with 60 slice per slab and 20% slice oversampling, with a reconstructed voxel size of 0.4 × 0.4 × 0.4 mm and FOV of 22 cm. Parameters for the PI TOF MRA sequence were TR 23 ms, TE 3.98 ms and flip angle of 18°. In total, 4 slabs were acquired with 36 slice per slab and 22% slice oversampling, with a voxel size of 0.4 × 0.4 × 0.7 mm. Acquisition time was 2.5 minutes for CS TOF and 7.5 minutes for PI TOF MRA.

### 2.2. Image Analysis

Image evaluation was performed by four examiners, two diagnostic neuroradiologists (FB and FT), one interventional neuroradiologist (LC) (each one with > 10 years of experience) and one radiology resident (GV). During image analysis examiners were blinded to the type of MRA sequence and to details regarding the endovascular procedures, except for the location of the coiled aneurysms.

In the first stage of analysis, we compared the performance of CS with PI TOF MRA in evaluating the degree of aneurysm occlusion as defined by the modified Raymond-Roy scale (Class I: complete obliteration; Class II: residual neck; Class IIIa: residual flow within the coil interstices; Class IIIb: residual flow along the aneurysm wall) [26]. Images of both MRA sequences were anonymized and randomly presented to the examiners in multiple sessions, taking care that every examiner evaluated only one type of sequence for each patient during each session.

In the second stage of analysis, we intended to evaluate differences in the visualization of the aneurysm parent vessel as well as of vessels originating from the aneurysm neck or coursing in close proximity to the aneurysm dome. Therefore, MIP images of both PI and CS TOF MRA of each patient were presented simultaneously to the examiners, who were asked first to evaluate whether one set of images were superior in the depiction of the aneurysm parent vessel and then to evaluate which allowed better visualization of other vessels adjacent to the aneurysm. The performance of CS TOF MRA was defined as “better”, “worse” or “equal” than PI TOF MRA on the basis of the examiner choice. The assessment of vessels depiction by the examiners was based on qualitative parameters, such as perceived signal intensity, edge sharpness, contrast and presence of susceptibility artifacts generated by the coils or stent.

### 2.3. Statistical Analysis

Continuous variables were described as median and interquartile range (IQR), whereas categorical variables were summarized by absolute and relative (%) frequency. The inter-modality agreement between sequences in the evaluation of aneurysm occlusion was assessed through Cohen’s kappa test on the entire set of readings, including all four examiners, whereas inter-rater reliability for the evaluation of aneurysm occlusion, parent vessel and adjacent vessels visualization was calculated using Fleiss’ kappa test. In both instances the following parameters were employed: κ  =  0–0.2 inadequate agreement, κ  =  0.21–0.4 slight agreement, κ  =  0.41–0.6 moderate agreement, κ  =  0.61–0.8 substantial agreement, κ  =  0.81–1.00 almost perfect agreement, and κ  =  1.00 perfect agreement. Comparison of CS and PI TOF MRA performance in depicting the aneurysm parent vessel and adjacent vessels in different subgroups was evaluated using Fisher’s exact test. All tests were 2-sided, and *p* < 0.05 was considered statistically significant. Statistical analysis was performed by using R version 3.6.1 (2019; The R Foundation for Statistical Computing, Vienna, Austria).

## 3. Results

### 3.1. Patient Population

We enrolled 22 patients in this study (72.7% females, Table 1), with a total of 22 intracranial aneurysms treated with either coil embolization or stent-assisted coiling. The median age at endovascular procedure was 63 years (IQR: 55 – 67 years) with a median follow-up time of 2.5 years (IQR: 0.9 – 4.7 years). In total, 5 (22.7%) patients underwent emergent aneurysm embolization after subarachnoid hemorrhage whereas the others underwent elective treatment. In total, 15 (68.2%) aneurysms were treated with coil embolization and seven (31.8%) with stent assisted coiling. The most frequent aneurysm location was the anterior communicating artery (36.4%), followed by the supraclinoid segment of the internal carotid artery (27.2%), M1-M2 bifurcation (13.6%), posterior communicating artery (9.1%), basilar artery (9.1%) and pericallosal artery (4.5%). Median aneurysm diameter was 7 mm (IQR: 6 - 10 mm).

### 3.2. Evaluation of Aneurysm Occlusion

**Figure 1:**
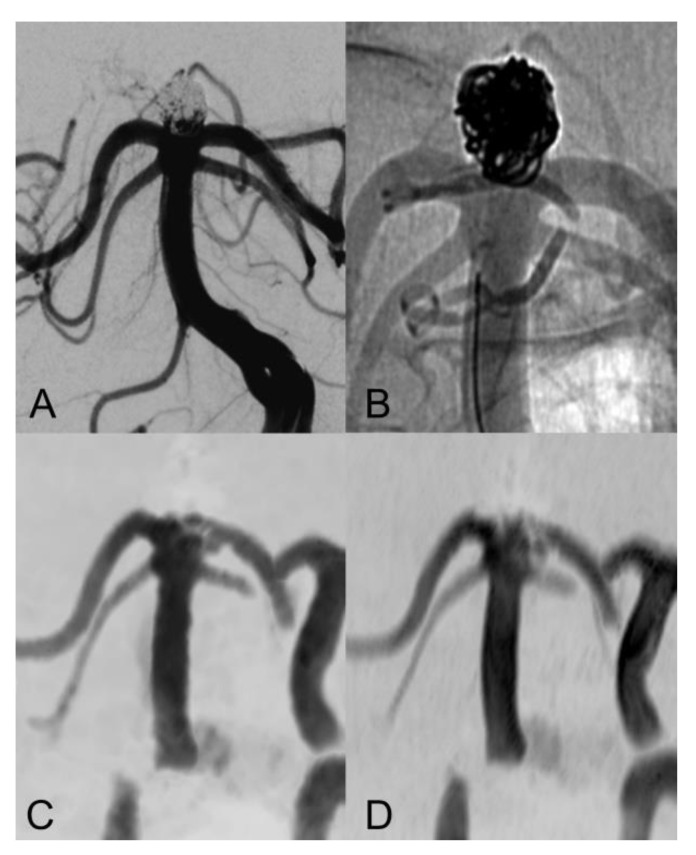
Anteroposterior view of vertebral artery digital subtraction angiography (DS) with (**A**) and without subtraction (**B**) after basilar-tip aneurysm coiling. Coronal MIP images of CS TOF (**C**) and PI TOF (**D**) showing complete occlusion of the aneurysm (class I). The interface between blood flow and coils is sharper on CS TOF images compared to PI TOF.

**Figure 2:**
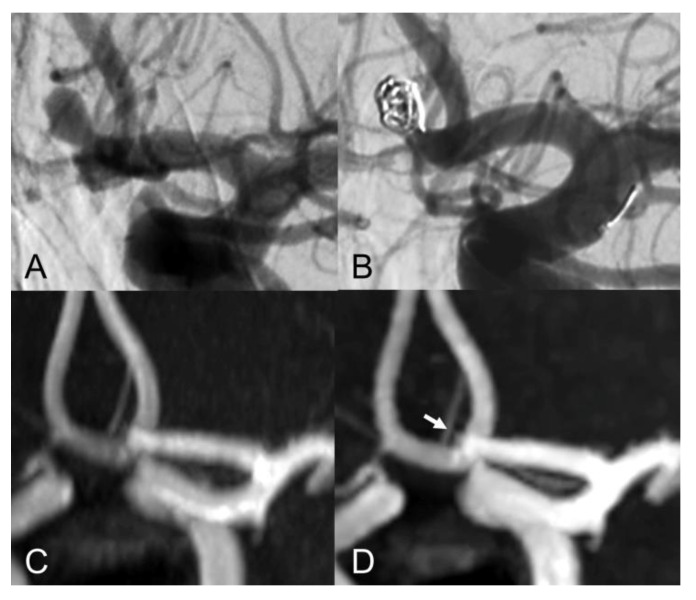
Lateral view of internal carotid artery DSA before (**A**) and after (**B**) anterior communicating artery aneurysm coiling. Coronal MIP images of PI TOF (**C**) showing complete occlusion of the aneurysm (class I) and CS TOF (**D**) better depicting a tiny neck residue (arrow), which was nonetheless correctly graded as class II only by the interventional neuroradiologist.

**Figure 3:**
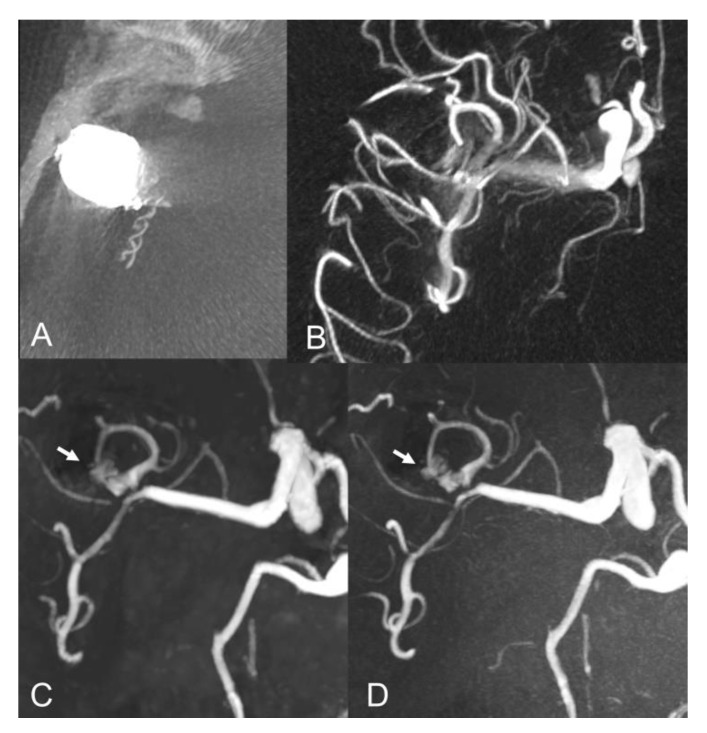
Baseline axial CT image (**A**) and vaso-CT (**B**) after stent-assisted coiling of a middle cerebral artery aneurysm. Follow-up axial MIP images of CS TOF (**C**) and PI TOF (**D**) showing residual flow (arrows) between the coils (class IIIb).

The evaluation of Class I - III coiled aneurysm occlusion status, with CS and PI TOF, is exemplified in Figure 1, Figure 2 and Figure 3.

The inter-modality agreement of CS and PI TOF MRA in the evaluation of aneurysm occlusion was perfect (κ  =  1.00, *p*  <  0.001) for all examiners except one, which classified one anterior communicating artery aneurysm as Class I on PI TOF images and as Class II on CS TOF images (Figure 2), leading, nonetheless, to an almost perfect inter-modality agreement (κ  =  0.90, *p*  <  0.001). The overall inter-modality agreement between the two techniques, considering all the readings by all the examiners (*n* = 88) was almost perfect (κ  =  0.98, *p*  <  0.001). No significant differences in inter-modality agreement were noted between patients who underwent coiling and stent-assisted coiling (κ  =  1.00 and κ  =  0.97, respectively). The overall inter-rater agreement was substantial (κ  =  0.70, *p*  <  0.001) and was almost identical for both CS TOF (κ  =  0.71, *p*  <  0.001) and PI TOF MRA (κ  =  0.68, *p*  <  0.001); 12 aneurysms (54.5%) were evaluated by all the examiners as completely occluded on both MRA techniques.

### 3.3. Evaluation of Adjacent Vessels

Considering the entirety of readings by all the examiners (*n* = 88), the depiction of aneurysm parent vessel in CS TOF images compared to PI TOF images was evaluated to be better in 10 (11.4%), equal in 76 (86.4%), and worse in 2 (2.3%) cases. CS TOF performed better in patients who underwent coil embolization without stent placement, showing better or equal visualization of aneurysm parent vessel compared to PI TOF in all readings (better in 9, 15.0%, and equal in 51, 85.0%). In patients treated with stent assisted coiling CS TOF was evaluated to depict aneurysm parent vessel better than PI TOF in 1 reading (3.6%) equal to PI TOF in 25 (89.3%) and worse than PI TOF in 2 (7.1%). This difference showed a trend towards significance (*p* = 0.051, Table 2). Inter-rater agreement for parent vessel evaluation was moderate (κ  =  0.47, *p*  <  0.001).

**Figure 4:**
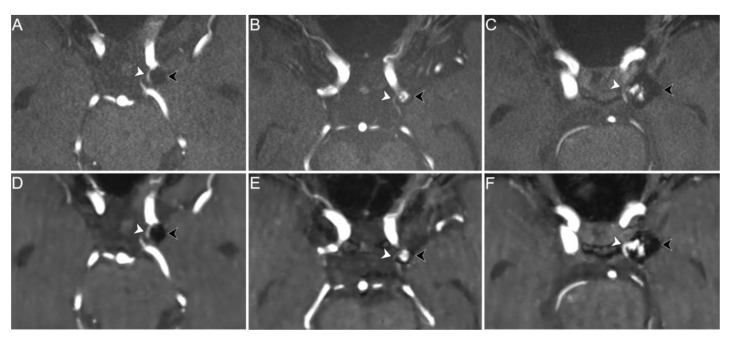
PI (upper row) and CS (lower row) TOF MRA images of three different patients exemplifying differences in performance between the two sequences in the depiction of parent vessel and vessels coursing in close proximity to the aneurysm dome. Axial PI TOF MRA (**A**) and CS TOF MRA (**D**) of a coiled aneurysm of the left supraclinoid internal carotid artery (black arrowheads) without signs of recanalization (class I); the proximal portion of the left anterior choroidal artery, which courses in close proximity to the aneurysm dome, is equally visualized on both sequences (white arrowheads). Axial PI TOF MRA (**B**) and CS TOF MRA (**E**) of a coiled aneurysm of the left supraclinoid internal carotid artery (black arrowheads) with recanalization of the aneurysm neck (class II); the proximal portion of the left anterior choroidal artery, which originates from the aneurysm neck, is better depicted by the PI TOF image (white arrowheads). Axial PI TOF MRA (**C**) and CS TOF MRA (**F**) of a coiled aneurysm of the left supraclinoid internal carotid artery (black arrowheads) with flow signal between the coils (class IIIa); the proximal portion of the left posterior communicating artery, which originates from the aneurysm neck, is better visualized in the CS TOF image (white arrowheads).

The depiction of vessels originating from the aneurysm neck or coursing in close proximity to the aneurysm dome in CS TOF images compared to PI TOF images was evaluated to be better in 8 readings (9.1%), equal in 70 (79.5%), and worse in 10 (11.4%) (Figure 4). No significant differences were found in CS TOF performance between patients with and without stent placement (*p* = 1, Table 2). Inter-rater agreement for the evaluation of vessels adjacent to the aneurysm was moderate (κ  =  0.42, *p*  <  0.001).

## 4. Discussion

Unenhanced MRA at 3T is effective at classifying coiled aneurysms as occluded or residually patent [14,15,27].

In this study, we found that CS TOF MRA has an almost perfect inter-modality agreement with PI TOF MRA for the evaluation of aneurysm occlusion in patients who underwent embolization with or without stent assistance, while reducing scan times from 7.5 to 2.5 minutes. Additionally, CS TOF MRA showed a high inter-rater agreement for the evaluation of aneurysm occlusion, which was almost identical to PI TOF MRA (κ  =  0.68 and κ  =  0.71, respectively). 

Our findings are in agreement with previous studies which showed comparable results between CS TOF and PI TOF MRA in the detection of a variety of cerebrovascular pathologies, including aneurysms, stenosis and arteriovenous shunts [21,22,23,24,25]. In particular, no significant differences were found between CS TOF and PI TOF MRA in the depiction of intact aneurysms, as well as in the measurement of neck, height and width of aneurysms [22]. In our study, only one case received a different occlusion classification in CS and PI TOF images, by the neurointerventionalist reader, who correctly appreciated a small neck remnant in CS TOF images, which was not apparent in the PI sequence (Figure 2). Therefore, while magnetic susceptibility artifacts due to the presence of the coil cast seem to have a comparable effect on both sequences in the majority of cases, we observed a sharper interface between blood flow and surrounding coils in some CS TOF images compared to PI TOF counterparts (Figure 1, Figure 2 and Figure 3).

When considering the evaluation of the parent vessel and vessels coursing close to the aneurysm dome, we found that CS TOF MRA performed equally or better than PI TOF MRA in 97.8% and 88.6% of cases, respectively. The only two patients in whom the parent vessel was better visualized on the PI TOF images were treated with stent assisted coiling for aneurysms originating from the anterior communicating artery and the pericallosal artery. Nonetheless, in 89.3% of readings the parent vessel was equally visualized on both sequences and in one case it was better depicted on CS TOF images. Previous studies showed that CS TOF MRA may result in worse visualization of smaller vessels compared to PI TOF MRA [21,28], therefore our results could be better explained by the small diameter of the stented vessels rather than the presence of susceptibility artifact due to the stent itself. This hypothesis is also supported by the observation that in the majority of cases in which CS TOF images showed a worse depiction of vascular structures adjacent to the aneurysm dome, the affected vessel was the anterior choroidal artery, which has a smaller diameter compared to the other examined vessels (Figure 4B,E). Nonetheless, further studies including a greater number of patients treated with stent placement are needed to assess the performance of CS TOF MRA in the evaluation of stented vessels.

Contrast enhanced (CE) MRA is faster than PI TOF MRA and while the majority of studies did not find a significant difference between the two sequences for the assessment of coiled aneurysms [7,14,15,17,29], some reports found that CE MRA offers better imaging of large aneurysm remnants and stented vessels [29,30]. However, the need for intravenous contrast medium injection may lead to adverse reactions and to gadolinium deposition in neuronal tissue, especially when serial examinations are required, as in patients with coiled intracranial aneurysm [31,32]. Additionally, the quality of CE MRA may be compromised by an incorrect acquisition timing, leading to suboptimal arterial enhancement and venous contamination. Therefore, CS TOF MRA may be a good first line approach in the follow-up of patients with coiled aneurysm, which allows for short acquisition times and is free from the potential risks of contrast media administration.

Recently, additional techniques, such as vessel-wall imaging and computational fluid dynamics, have been proposed for the evaluation of coiled aneurysms. These methods offer additional information on the processes leading to aneurysm formation and growth and may be helpful in identifying patients at greater risk of aneurysm recurrence after coiling [33,34].

Our study has some limitations. First, the small sample size, which will require larger studies to confirm the validity of our results in clinical practice. Second, the diagnostic accuracy of both sequences was not compared to DSA. However, our goal was to evaluate CS TOF MRA performance compared to PI TOF MRA, whose high sensitivity and specificity in the detection of residual aneurysm has already been established [7,8,9]. Third, none of the patients included in this study underwent parent vessel reconstruction using flow-diverting stents; further studies are needed to evaluate the performance of CS TOF MRA in patients treated with this technique. Publicly available databases, with data contributed by several centers, will be essential to validate the routine use of CS TOF MRA in clinical practice.

## 5. Conclusions

In our preliminary study, we found that CS TOF MRA, with scan times of only 2.5 min, was comparable to PI TOF MRA (with scan times of 7.5 min) for assessing the occlusion status of coiled intracranial aneurysms as well as for visualizing the parent vessel and vascular structures adjacent to the aneurysm dome. Short scan times reduce the risk of motion artifacts, allowing examination of uncooperative patients, and increase patient comfort, which is particularly relevant for those needing frequent follow-up scans. Finally, the benefits of CS TOF MRA are also associated with an increased patient throughput and a resulting reduction in costs, making this sequence a potential replacement for PI TOF MRA as a first line follow-up examination in patients with intracranial aneurysms treated with coil embolization. Further multicenter studies, supported by scientific Neuroradiological Societies, recruiting larger samples of patients, will be needed to validate our results in clinical practice.

## Figures and Tables

**Table 1:** Demographic, clinical and procedure data.

Sex (female)	16 (72.7%)
Age (years)	63 (55–76)
Follow-up time (years)	2.5 (0.9–4.7)
Subarachnoid hemorrhage	5 (22.7%)
Stent placement	7 (31.8%)
Aneurysm location	
ACA	8 (36.4%)
Supraclinoid ICA	6 (27.2%)
M1-M2 bifurcation	3 (13.6%)
PCA	2 (9.1%)
Basilar artery	2 (9.1%)
Pericallosal artery	1 (4.5%)
Aneurysm diameter (mm)	7 (6–10)

Categorical variables are expressed as absolute and relative (%) frequencies, continuous variables as median and interquartile range (IQR). ACA Anterior communicating artery; ICA Internal carotid artery; PCA Posterior communicating artery.

**Table 2:** Comparison of CS vs PI TOF MRA performance in depicting aneurysm parent vessel and adjacent vessels.

	Total Readings (*n* = 88)	Coil Embolization	Stent-Assisted Coiling	*p*-Value
Parent vessel depiction				0.051
Better	10 (11.4%)	9 (15.0%)	1 (3.6%)	
Equal	76 (86.4%)	51 (85.0%)	25 (89.3%)	
Worse	2 (2.3%)	0 (0.0%)	2 (7.1%)	
Adjacent vessels depiction				1
Better	8 (9.1%)	6 (10.0%)	2 (7.1%)	
Equal	70 (79.5%)	47 (78.3%)	23 (82.1%)	
Worse	10 (11.4%)	7 (11.7%)	3 (10.7%)	

## Data Availability

The data presented in this study are deposited in Zenodo repository (https://zenodo.org) and are accessible with the following link: https://doi.org/10.5281/zenodo.6654502.
